# Intractable Chronic Vulval Ulceration Presenting as Immune Reconstitution Inflammatory Syndrome in a Treatment-Failure Patient: A Case Observation

**DOI:** 10.1155/2011/896964

**Published:** 2011-09-22

**Authors:** Christine Katusiime, Ponsiano Ocama, Andrew Kambugu

**Affiliations:** ^1^Department of Prevention, Care and Treatment, Infectious Diseases Institute, Makerere University College of Health Sciences, Kampala, Uganda; ^2^Department of Medicine, Makerere University College of Health Sciences, Kampala, Uganda

## Abstract

HIV-1 treatment-failure patients are increasingly being initiated on second-line antiretroviral therapy. The case we describe is of a treatment-failure patient who developed intractable chronic vulval ulceration presenting as immune reconstitution inflammatory syndrome (IRIS), following complete viral suppression with second-line highly active antiretroviral treatment (HAART). To the best of our knowledge, this is the first reported case of intractable vulval ulceration IRIS in an HIV-1 treatment-failure patient.

## 1. Introduction

IRIS manifesting as genital ulcer disease (GUD) has been documented in some reports [1, 2]. GUD as an IRIS event in treatment-failure patients on the contrary has not been documented. We describe a treatment-failure patient who following complete viral suppression developed chronic vulval ulceration despite thorough and extensive investigations.

## 2. Case Presentation

A 35-year-old HIV-positive woman presented to the Infectious Diseases Institute, Kampala, in February 2005 with World Health Organization (WHO) stage III disease having presented with a history of >10% weight loss and recurrent upper respiratory tract infections (URTIs). Her nadir CD4+ count was 9 cells/*μ*L. She was initiated on HAART—with a fixed dose combination of lamivudine 150 mg/stavudine 40 mg/nevirapine 200 mg (Triomune-40) one tablet twice daily and cotrimoxazole prophylaxis to which she reported 100% adherence. After 1.5 years on HAART, she defaulted on therapy for a period of 4 months. She subsequently began receiving care from another HIV treatment centre where she had a dual drug switch to tenofovir, emtricitabine (Truvada), and nevirapine, to which she complied with 100% adherence. She continued treatment at that centre for 2 years. During this period she had an initial rise in CD4+ cell count to a peak of 389 cells/*μ*L which subsequently started decreasing gradually both in absolute count and percentage. 

At this juncture, she was referred back to our institute where she was found with a CD4+ count of 30 cells/*μ*L (2%). A decision was made to maintain her on truvada and nevirapine following adherence counseling. A repeat CD4+ count 8 months later showed a further decline in the absolute CD4+ count and percentage to 12 cells/*μ*L (1%), while still on truvada and nevirapine. She had a detectable viral load of 36,795 copies/mL. She was then switched to abacavir, zidovudine/lamivudine (Combivir), and lopinavir/ritonavir (Alluvia) basing on possible development of K103N and M184V mutations. A resistance profile could not be done due to the high costs involved. A repeat laboratory profile four months later showed immunological improvement with a rise in CD4+ count and percentage to 75 cells/*μ*L (4%) and an undetectable viral load of <400 copies/mL. 

The second-line HAART was well tolerated with sustained viral suppression of <400 copies/mL and immunological improvement to 209 cells/*μ*L (9%) six months later. Four months after commencing second-line HAART and achieving complete viral suppression, she presented with complaints of vulval ulcers. Genital examination revealed a 4 cm superficial right vulval ulcer. There were no other significant findings on systemic examination. Cryptococcal antigen and Syphilis serology (Venereal Disease Research Laboratories and *Treponema pallidum* haemagglutination assay) were negative. Herpes simplex virus-2 serology could not be done due to the high costs involved. Swabs from the vulval ulcer and cervix were unremarkable. Two biopsies revealed nonspecific chronic inflammation. 

Treatment with oral acyclovir, famciclovir, ampiclox (ampicillin/cloxacillin), cloxacillin, ceftriaxone, fluconazole, metronidazole, ciprofloxacin, doxycycline, nonsteroidal anti-inflammatory drugs (NSAIDs), and topical hydrocortisone cream was unsuccessful. A routine of oral prednisolone 40 mg once daily given over ten days which was gradually tapered off over a period of 4 weeks was repeated quarterly with complete resolution approximately three years later. Chlorhexidine and cetrimide (Hibitane) soaks were also applied.

## 3. Discussion

GUD-IRIS has been shown to significantly peak in the first month after HAART commencement [1]. Acute GUD-IRIS has been commonly described as primarily syphilitic and herpetiform [1, 3]. Adenovirus, cytomegalovirus, M. *tuberculosis*, and M. *avium* complex have been implicated in chronic GUD-IRIS in addition to herpes simplex [2, 4, 5]. 

IRIS may manifest as infectious or autoimmune clinical events predominantly occurring in the first 8 weeks following HAART initiation with the exception of Graves' disease which occurs at a median time of 2 years [6–9]. Chronic genital ulceration-IRIS has been described twice before [2, 5]. On the contrary, chronic genital ulceration IRIS lasting over two years is rare and has been described only once before [2]. Idiopathic chronic vulval ulceration IRIS, however, in reconstituting HIV treatment failure patients has not been described before, and, to the best of our knowledge, this is the first reported case of idiopathic chronic vulval ulceration-IRIS in a reconstituting HIV treatment-failure patient. 

In our patient, the cause of intractable chronic vulval ulceration for over three years was idiopathic, following extensive microbiological, histopathological investigations and extensive treatment—with the use of antibiotics, antivirals, NSAIDs, and steroids. It is therefore imperative that clinicians are aware of chronic intractable genital ulceration-IRIS that defies extensive treatment and investigations when HIV treatment-failure patients undergo immune reconstitution.
